# Seasonal Variation of Bacterial Diversity Along the Marine Particulate Matter Continuum

**DOI:** 10.3389/fmicb.2020.01590

**Published:** 2020-07-21

**Authors:** Mireia Mestre, Juan Höfer, M. Montserrat Sala, Josep M. Gasol

**Affiliations:** ^1^Department of Marine Biology and Oceanography, Institut de Ciències del Mar (ICM-CSIC), Barcelona, Spain; ^2^Centro de Investigación Oceanográfica COPAS Sur-Austral, Departamento de Oceanografía, Universidad de Concepción, Concepción, Chile; ^3^Centro FONDAP de Investigación en Dinámica de Ecosistemas Marinos de Altas Latitudes (IDEAL), Universidad Austral de Chile, Valdivia, Chile; ^4^Escuela de Ciencias del Mar, Pontificia Universidad Católica de Valparaíso, Valparaíso, Chile; ^5^Centre for Marine Ecosystem Research, School of Sciences, Edith Cowan University, Joondalup, WA, Australia

**Keywords:** bacterial diversity, seasonal dynamics, marine particles, temperate sea, oligotrophic, coastal

## Abstract

Seasonal dynamics of ocean prokaryotic communities in the free-living fraction have been widely described, but less is known about the seasonality of prokaryotes inhabiting marine particles. We describe the seasonality of bacterial communities in the particulate matter continuum by sampling monthly over two years in a temperate oligotrophic coastal ecosystem and using a serial filtration (including six size-fractions spanning from 0.2 to 200 μm). We observed that bacterial communities in the particulate matter continuum had annual changes following harmonic seasonal oscillations, where alpha, beta, and gamma diversity increased during the warm period and decreased during the cold period. Communities in each size-fraction changed gradually over time, being the communities in larger size-fractions the ones with stronger annual changes. Annual community changes were driven mainly by day length and sea surface temperature, and each size-fraction was additionally affected by other variables (e.g., smaller size-fractions by dissolved PO_4_ and larger size-fractions by turbidity). While some taxonomic groups mantained their preference for a given size fraction during most of the year, others varied their distribution into different size fractions over time, as e.g., SAR11, which increased its presence in particles during the cold period. Our results indicate that the size-fractionation scheme provides novel seasonal patterns that are not possible to unveil by analyzing only free-living bacteria, and that help to better understand the temporal dynamics of prokaryotes.

## Introduction

Ecological communities are dynamic (e.g., Magurran et al., 2010) and, in particular, aquatic microbial communities are known to vary over different temporal scales (e.g., Fuhrman et al., 2006; Kara and Shade, 2009; Gilbert et al., 2012; Jones et al., 2012; Hatosy et al., 2013). Annual surveys demonstrate that the structure of marine free-living (FL) bacterial communities can be predicted from ocean conditions (Fuhrman et al., 2006), and the annual succession of FL ocean surface bacterioplankton communities has been well described in temporal surveys performed in tropical, temperate, and polar regions (reviewed in Bunse and Pinhassi, 2017). In temperate seas, the most studied ones, the dynamics of FL bacteria is governed by changes in day length, temperature, nutrients, and chlorophyll *a* (Pinhassi et al., 2006; Gilbert et al., 2009; Andersson et al., 2010). In these waters, typical summer water column stratification is associated with communities dominated by Cyanobacteria, *Roseobacter*, SAR86, and SAR11 (Schauer et al., 2003; Alonso-Sáez et al., 2007; Lindh et al., 2015). During winter and spring the mixing of the water column promotes bacterial communities dominated by *Flavobacteria*, *Roseobacter*, and some *Gammaproteobacteria* (Teeling et al., 2012; Buchan et al., 2014; Taylor et al., 2014). However, all these studies have described only the temporal changes of FL bacteria, and much less is known on the annual variability of the bacterial communities attached to particles.

Free-living and particle-attached (ATT) marine bacteria are known to be taxonomically different (e.g., DeLong et al., 1993; Grossart et al., 2005; Ortega-Retuerta et al., 2013) and represent two distinct lifestyle strategies: FL microorganisms tend to be adapted to low substrate concentrations (Satinsky et al., 2014), to have smaller genomes (Smith et al., 2013) and exhibit higher motility (Mitchell et al., 1995; Fenchel, 2001; Grossart et al., 2001). In contrast, ATT bacteria are often larger than FL bacteria (Alldredge et al., 1986; Simon et al., 2002), form denser communities of cells (Simon et al., 2002), and have higher production (Kirchman and Mitchell, 1982) and respiration rates (Grossart et al., 2007). ATT bacteria also have higher extracellular enzyme activities (Karner and Herndl, 1992; Smith et al., 1992), and are able to hydrolyze more recalcitrant substrates (Grossart and Simon, 1998; Kiørboe and Jackson, 2001; Kiørboe et al., 2002). Phytoplankton and zooplankton are the main source of pelagic particles where marine bacteria attach (reviewed in Simon et al., 2002) and both functional groups display a clear seasonal cycle in temperate seas (e.g., Siokou-Frangou, 1996; Calbet et al., 2001; d’Alcalà et al., 2004; Cabrini et al., 2012; Nunes et al., 2018). We would hypothesize thus, that bacterial communities attached to particles should exhibit a seasonal cycle, whose temporal dynamics may probably be different to that of FL bacteria.

Particulate matter is present in aquatic environments in a continuum of sizes (Azam et al., 1993; Verdugo et al., 2004). Thus, the use of a multiple size-fractionation approach which separates the continuum of sizes of the plankton particulate matter into a defined number of discrete size-fractions will provide a more exhaustive description of the bacterial diversity and community structure than separating bacteria simply using the dichotomy FL vs. ATT (Mestre et al., 2017a). For example, multiple size-fractionations revealed that particles of different size (i.e., in this case six size-fractions, ranging from 0.2 to 200 μm) harbor different bacterial communities, richness can be six times higher in the larger size-fractions than in the smaller ones, and taxonomic groups can be better described by their preference for a given size-fraction, instead of classifying them simply as FL vs. ATT bacteria (Mestre et al., 2017a). Recent studies have described the spatial variability of the communities in different size-fractions from the coast to the open ocean and from the surface to the deep ocean (Mestre et al., 2017b, 2018), but we still know very little about how bacterial diversity and community structure in the continuum of particulate matter vary temporally. Up to date, only three studies, all in temperate areas, have explored the temporal dynamics of FL vs. ATT communities, and those studies were performed in a coastal lagoon (Mohit et al., 2014), an estuary (Selje and Simon, 2003), and an eutrophic coastal site (Yung et al., 2016). In two of the cases (Selje and Simon, 2003; Mohit et al., 2014), the authors used only one filter to separate the FL from the attached bacterial communities, thus, overlooking the possible differences in communities associated to particles of distinct size ranges. In a more comprehensive sampling, Yung et al. (2016) analyzed the bacterial communities present in four different size-fractions but approached the community as a whole and did not describe the variations over time of the more abundant taxonomic groups associated with particles. These studies showed differences in the composition of FL and ATT communities and clear temporal trends, but the seasonality and the interannual recurrence were not unveiled because their sampling lasted for one year or less. Therefore, we still do not know: (1) whether the bacterial communities and individual taxonomic groups associated to different size-fractions have seasonality; (2) whether the seasonal patterns described are repeatable and extendable to a longer period (i.e., if there is interannual recurrence also in the larger size fractions); and (3) which environmental factors drive these patterns.

We characterized monthly the diversity of bacteria in six different size-fractions (ranging from 0.2 to 200 μm), in the Blanes Bay Microbial Observatory (BBMO), over two years, to determine whether bacteria in the different size-fractions display repeatable changes throughout the year at both individual taxonomic groups and community levels, and to identify the key drivers influencing FL and ATT bacteria seasonality. The BBMO is an oligotrophic coastal system in the NW Mediterranean sea with seasonal dynamics typical of a temperate region and relatively unaffected by human influence (Gasol et al., 2012). Bacterial richness at the BBMO is higher for the larger size-fractions (Mestre et al., 2017a) and given the seasonal changes in phytoplankton (Gasol et al., 2016; Nunes et al., 2018), pico- and nano-plankton (Giner et al., 2019), and zooplankton (Calbet et al., 2001) in the area, we hypothesize that the dynamics of bacterial diversity will be strongly structured by size-fraction, and that we will be able to detect clear and different seasonal patterns for each size-fraction.

## Materials and Methods

### Study Area, Sampling, and Environmental Conditions

Samples were taken monthly between June 2011 and June 2013 at the BBMO, an oligotrophic coastal station (20 m depth) placed 0.5 miles offshore (41°40’N, 002°48’E) in the NW Mediterranean Sea, which has regularly been sampled for microbial ecology studies during the last decades (Gasol et al., 2012, 2016). Surface water (0.5 m depth) was taken and pre-filtered through a 200-μm mesh net and transported to the laboratory in darkness. For bacterial diversity analyses, a total of 10 L was filtered using a peristaltic pump at very low speed and pressure, and sequentially through 10, 5, 3, 0.8 and 0.2 μm pore-size polycarbonate filters of 47 mm diameter (Millipore, Billerica, MA, United States). The first year the system incorporated a mesh net of 20 μm, whereas the second year a 20 μm pore-size polycarbonate filter of 47 mm diameter (GE Water and Process Technologies. Trevose, United States) was added, increasing by one the number of size-fractions. We minimized clogging and disaggregation processes by prefiltering all the samples through 200 μm, using very low vacuum pressure, and changing the filters when the flow slowed down (usually the 0.2 and 0.8 μm pore-size filters were replaced at least once per filtration). All the filters of the same pore-size were pooled to be processed together as one single sample. All filters were stored immediately at −80°C until extraction. The size-fractions were defined as: 0.2–0.8; 0.8–3.0; 3.0–5.0; 5.0–10; 10–20 and 20–200 μm. In parallel, a set of environmental conditions [day length, temperature, salinity, secchi disk depth, chlorophyll *a*, inorganic nutrients, total organic carbon (TOC), particulate organic carbon (POC), bacterial activity, and bacterial abundance] were measured. Methods for determination of these environmental parameters can be found in Supplementary Methods S1.

### DNA Extraction, Sequencing, and Sequence Processing

The DNA was extracted as described in Massana et al. (1997) and hypervariable regions V1-V3 16S DNA tags were PCR amplified with the primers 28F/519R (specific for bacteria, and not for archaea). It has been recently reported (Dadon-Pilosof et al., 2017) that this primer pair can overestimate or underestimate relative abundances of some taxonomic groups when compared to primers 515F-926R (Parada et al., 2016). Still, in our study the biases would apply equally to all samples and a general overestimation or underestimation of relative abundances of certain groups would not interfere with their variability along the size fractions or over time and thus with the temporal patterns we describe. The PCR products were 454 GS FLX+ pyrosequenced by Research and Testing Laboratory (Lubbock, TX, United States)^1^. Reads from 150 to 600 bp were quality checked (Phred quality average > 25) by using a 50 bp sliding window in QIIME (Caporaso et al., 2010). Pyrosequencing errors were reduced with Denoiser and the reads were clustered into OTUs with a 97% similarity threshold with UCLUST within QIIME. Chimeras were detected with ChimeraSlayer (Haas et al., 2011) and SILVA108 was used as a reference database, in MOTHUR (Schloss et al., 2009). Taxonomy assignment was done using SILVA Incremental Aligner (SINA v1.2.11). Unwanted OTUs (eukaryotes, chloroplast, mitochondria or OTUs with less than 5 sequences in total) were removed. The samples were randomly subsampled to the number of reads present in the sample with the lowest number of reads (*n* = 1,000).

### Data Analysis

Statistical analyses and graphs were done in R^2^ using the packages *vegan* (Oksanen et al., 2017), *simba* (Jurasinski and Retzer, 2015), and *minpack.lm* (Elzhov et al., 2016). The OTUs were grouped at phylum, class and genera level.

To elucidate how community assembly within each size-fraction varies along time, Bray–Curtis distances were calculated between a given size-fraction in January 2013 and the same size-fraction at other sampling dates. January 2013 was selected as a reference because it was the sample with higher diversity. The disimilarities of community assembly among all samples were also calculated with Bray–Curtis distances, and distances were visualized by non-metric multidimensional scaling (nMDS) analysis. Finally, statistical differences between size-fraction, month and year were explored with a permutational multivariate analysis of variance test (PERMANOVA), with the *adonis* function (R *vegan* package). The environmental variables that most influenced community composition were determined with the function *bioenv* (R package *vegan*) using the OTUs table and the environmental matrix combined. The environmental matrix included: day length, sea surface temperature, salinity, secchi disk depth, chlorophyll *a*, nutrients, POC, TOC, bacterial activity, and bacterial abundance. The influence of environmental variables on community assembly was visualized with a distance-based redundancy analysis (dbRDA), performed with function *capscale* (R package *vegan*).

To describe how bacterial diversity varied in the context of the six size-fractions over time, the true alpha (the average richness among the six size-fractions), the true gamma (the total richness of the six size-fractions) and the true beta diversity (the taxonomic differentiation between the six size-fractions) were calculated for each month with the R package *simba* following Tuomisto (2010).

The annual components of the variables day length, sea surface temperature, average alpha-, beta-, and gamma-diversity were analyzed by a harmonic analysis performed with the Levenberg-Marquardt algorithm for solving nonlinear least-squares problems, using the function *nls.lm* of the R package *minpack.lm* (Elzhov et al., 2016), and where the error for the estimated unkown variables was determined by the Hessian matrix. Data was adjusted to the following trigonometric equation:

$$Y=b1+b2\cdot \cos\left(\frac{2\pi }{365}\cdot t+b3\right)$$

Where *Y* is the variable analyzed, *b*1 is the annual mean, *b*2 the amplitude, *b*3 the diphase (ordinal date of the annual maximum), 365 the period, and *t* the ordinal date, ranging from 1 to 365.

We selected the most abundant taxonomic groups, i.e., those that represented >1% of the total abundance, at least once, in at least one size-fraction (17 taxonomic groups in total). The remaining taxonomic groups were considered “rare” and pooled together as “other bacteria”. To evaluate whether these taxonomic groups maintained or shifted their preferences for a given size-fraction throughout the year, we determined an “Heterogeneous Distribution Index” (HDI), which was calculated as follows: for each taxonomic group, the relative abundances of all size-fractions and months were averaged (named as “annual average” of a particular taxonomic groups). Taking the annual averages of a given taxonomic group as a reference, we calculated the deviations that occurred every month and in each size-fraction from that annual average (the absolute difference between a given value and the annual average). Then, the sum of the absolute deviations of all size-fractions in each month for a given taxonomic group was calculated. This relative value, which we named HDI, has no units and is a relative measure, being low when all distributions are similar, high when they are very distinct, and equal to 1 if all distributions are identical. This value could then be averaged across all months for a given taxonomic group (annual HDI for a given taxonomic group), or across all taxonomic groups for a given month (monthly HDI for the whole community). For more details, see Supplementary Methods S2.

## Results

This study was performed at the BBMO, an oligotrophic temperate coastal site, characterized by a strong seasonal forcing, with warm summers and relatively cold winters. This seasonal variability was well represented during this 2-year study (Supplementary Figure S1): Day length and sea surface temperature presented a continuous and harmonic variation along the year, with an annual maximum of day length in June (average 15.2 h) and an annual minimum in December (average 9.2 h), and an annual maximum of sea surface temperature in August (24.1°C) and an annual minimum in February-March (12.5°C). These values were not distinct from previous descriptions including more than 10 years of data (e.g., Gasol et al., 2016).

### Temporal Variability of the Bacterial Communities

**FIGURE 1:**
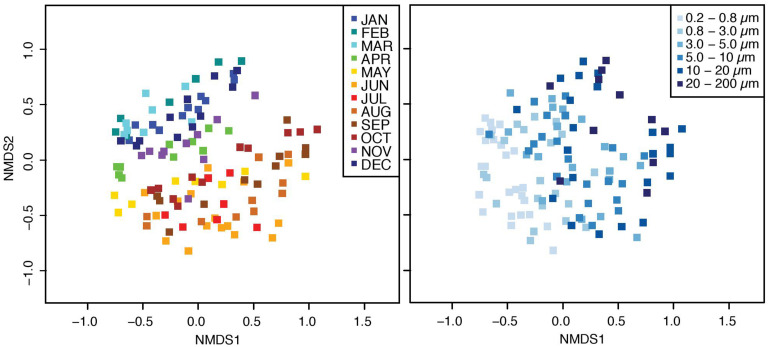
nMDS ordinations representing the Bray–Curtis distances between bacterial communities. Distances were calculated from the rarefied OTU table. Samples are color-coded depending on the month **(left panel)** and size-fraction **(right panel)**.

The bacterial communities were structured in a gradient along the size-fractions and over time, according to a nonmetric multidimensional scaling plot (Figure 1). Further, the nMDS showed that communities were separated into two main groups, corresponding to two main periods: from November to April (colder months), and from May to October (warmer months). A PERMANOVA test confirmed that the observed differences were statistically significant: communities were different according to the factors “size-fraction” (communities differed from one size-fraction to the other, PERMANOVA_bySize–fraction_ *R*^2^ = 0.279, *p* < 0.001, *n* = 120), “month” (communities differed from one month to the other, PERMANOVA_byMonth_ *R*^2^ = 0.275, *p* < 0.001, *n* = 120), and among two major clusters (communities from colder months were different from those of warmer months, PERMANOVA_byCluster_ *R*^2^ = 0.095, *p* < 0.001, *n* = 120).

**FIGURE 2:**
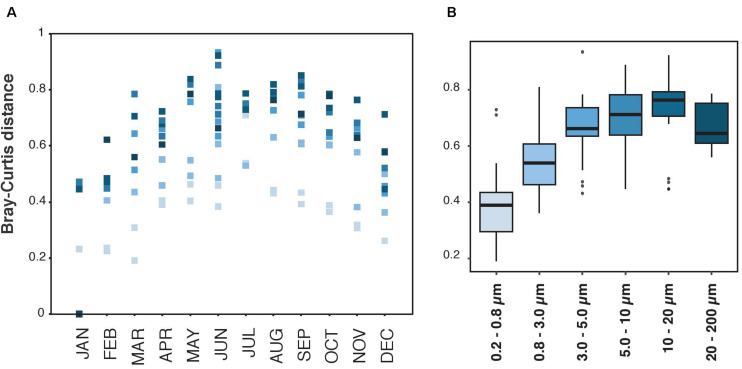
Bray-Curtis distances measured between January 2013 and the other months, and for each size-fraction separately. **(A)** Representation of Bray-Curtis values along time, from January to December. The dots are colored by size-fraction as they are in panel **(B)**. **(B)** Representation of Bray-Curtis values of each size-fraction. The upper and lower lines of each box-plot correspond to the 1st and 3rd quartile of the distribution of values. The median values are shown with horizontal black wide lines.

The communities varied temporally, and this variation was gradual through the year (Figure 2A). Still, the magnitude of this gradual variation was distinct in each size-fraction, being the smaller size-fractions the ones with less variation of community composition through the year (Figure 2B). In general, the magnitude of this variability increased from the smallest towards the larger size-fractions, and the size-fraction with higher seasonal variability was the 10–20 μm size-fraction. However, a decrease in the magnitude of this variability was observed in the largest size-fraction (20–200 μm, Figure 2B).

**FIGURE 3:**
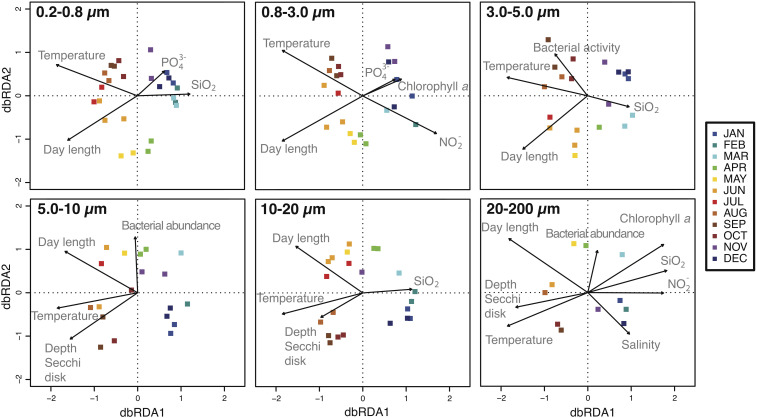
Distance-based redundancy analysis (dbRDA) representing samples over the year (square dots) and the environmental parameters that best explain the distribution of the communities (black arrows). Each panel corresponds to the different size-fractions. See section “Materials and Methods” for details.

The variables that best predicted the temporal changes in community composition of the overall dataset were sea surface temperature (*r* = −0.92) and day length (*r* = −0.82) (Supplementary Table S1). In addition, temperature and day length separated samples mainly into two main groups, determined by the axis dbRDA_1_ (Supplementary Figure S2). The dbRDA performed with each size-fraction separately showed that bacterial community composition of a particular size-fraction was driven by sea surface temperature and day length, but also by a particular combination of environmental factors (Figure 3). As a general trend, we observed that turbidity (i.e., Secchi depth) played an important role in the larger size-fractions (i.e., 5.0–10, 10–20, and 20–200 μm), whereas PO_4_ concentration was more relevant in the smaller size-fractions (i.e., 0.2–0.8 and 0.8–3.0 μm) (Supplementary Table S2).

**FIGURE 4:**
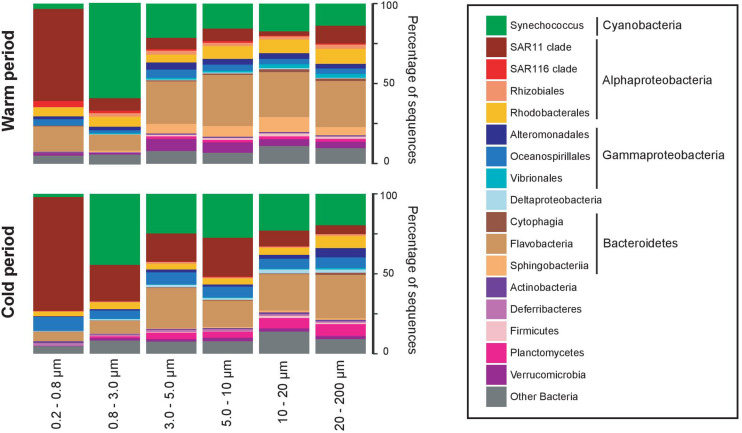
Average taxonomic composition of the cold (November–April) and warm (May–October) periods, and in each size-fraction. Community composition is expressed as percentage of the total number of sequences in each size-fraction (for more details see section “Materials and Methods”). Only taxonomic groups with >1% in abundance are represented, and the remaining taxonomic groups are pooled together as “Other bacteria”.

Considering the clustering configuration of the bacterial communities observed in the nMDS and the dbRDA analyses, we defined 2 major assemblies of communities, corresponding to the warm-period communities and the cold-period communities. Both periods presented in general the same dominant taxa, but different community assembly (Figure 4). *Synechococcus* sp., SAR11 and *Flavobacteria* dominated both periods, where SAR11 and *Synechococcus* sp. had preference for smaller size-fractions (0.2–0.8 μm and 0.8–3.0 μm, respectively), and *Flavobacteria* had preference for larger size-fractions. *Synechoccoccus* and *Flavobacteria* were more abundant during the warm period, and SAR11 during the cold period. The warm period was also characterized by the higher presence of *Rhizobiales, Rhodobacterales*, and *Sphingobacteriia* (with preference for larger size-fractions), whereas *Planctomycetes* was more abundant during the cold period (with preference for larger size-fractions).

### Modeling Diversity and Environmental Variables Over Time

We observed a large variability in richness (number of OTUs) between size-fractions and over time (Supplementary Figure S3). As a general pattern, we observed that richness increased with increasing size-fraction (Supplementary Figure S4). In addition, we observed an increase of diversity (alpha, beta and gamma diversity) during the warm period, and a decrease of diversity (alpha, beta, and gamma diversity) during the cold period (i.e., the average number of species, the total number of species and the global community differentiation among the size-fractions of a given month increased during the warm period, and decreased during the cold period) (Supplementary Figure S5).

**FIGURE 5:**
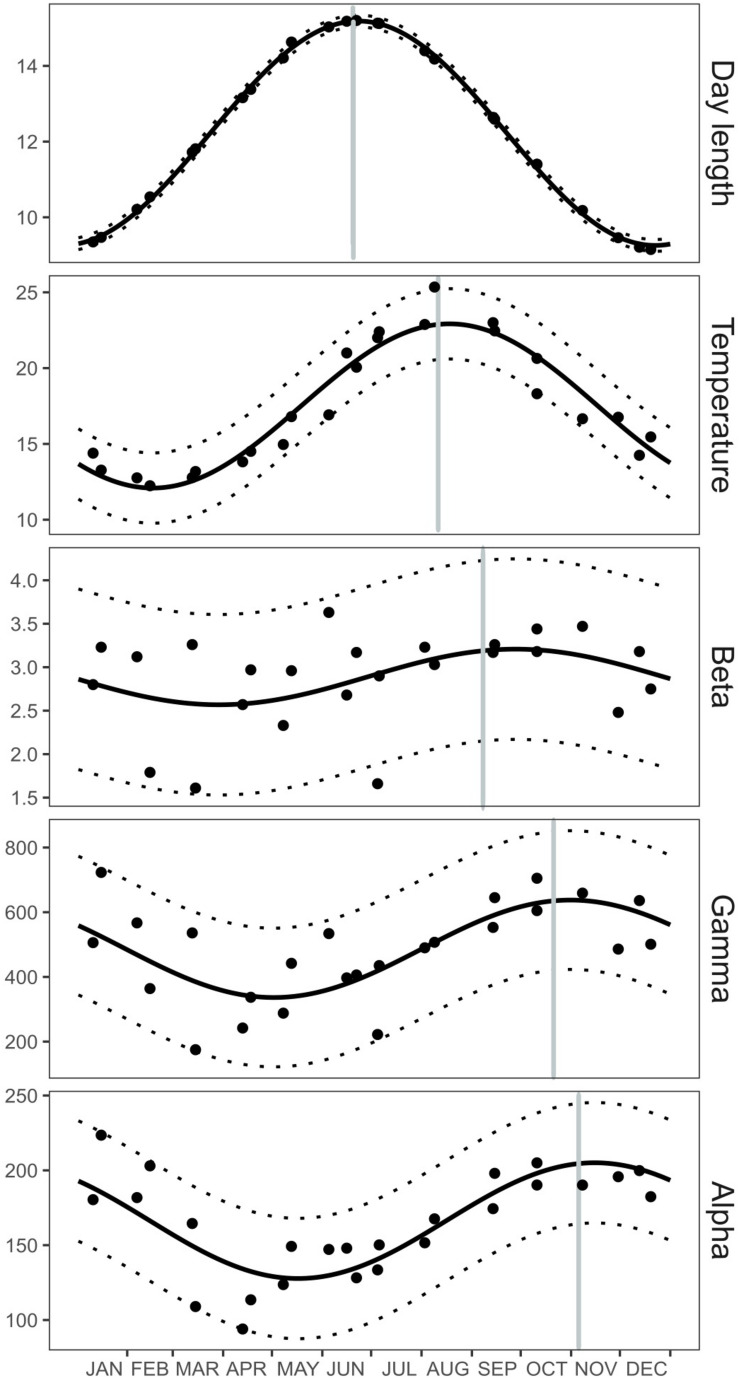
Seasonal cycle of day length, sea surface temperature, average alpha-, beta-, and gamma diversity. Black dots represent the data registered at Blanes Bay from June 2011 to June 2013. The solid black lines correspond to model-based values obtained after applying the results from the harmonic analysis, while the dotted black lines represent the 95% confidence interval of the predicted values. Vertical gray lines portray the ordinal date of the annual maximum of each seasonal cycle (*b*3) according to the harmonic analysis. Details about the fitted parameters of the harmonic analyses and their errors can be found in Supplementary Table S3.

The temporal dynamics of water temperature, day length, average alpha, beta, and gamma diversity were studied using a harmonic analysis of each time series. The harmonic analysis shows that the seasonal cycle of each variable was different (Supplementary Table S3) with day length peaking earlier in the year (June) and being followed by water temperature in August, beta diversity in September (i.e., larger differences between particles), gamma diversity in October (i.e., higher diversity within each size fraction), and alpha diversity in November (i.e., higher total diversity) (Figure 5). The sequence shown by these similar seasonal cycles suggests the possibility of a mechanistic relationship linking these variables.

### Temporal Variability of the Bacterial Dominant Taxa

Seventeen taxonomic groups had abundances >1% and dominated the community assembly during the two years, but their relative abundances were highly variable among size-fractions and over time (Supplementary Figure S6). We classified these taxonomic groups into four categories, considering their preference for small or large size-fractions (structure across size-fractions) following Mestre et al. (2017a): (a) taxonomic groups enriched in the small size-fractions (for example SAR11); (b) taxonomic groups enriched in the smaller size-fractions, but depleted or absent in the smallest one (0.2–0.8 μm, such as *Synechococcus*); (c) taxonomic groups that did not present enrichment in relation with size-fraction (e.g., *Deltaproteobacteria*); and (d) taxonomic groups enriched in the larger size-fractions (e.g., *Flavobacteria*).

**FIGURE 6:**
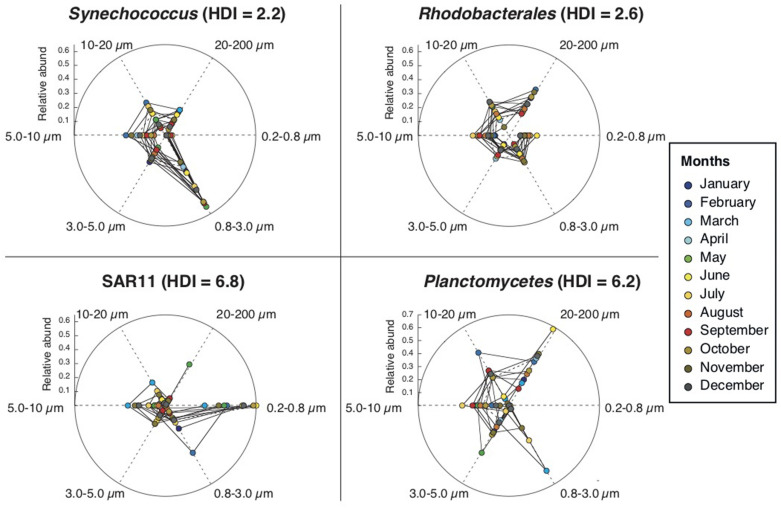
Examples of taxonomic groups with low (*Synechococcus* and *Rhodobacterales*) and high (SAR11, *Planctomycetes*) Heterogeneous Distribution Index (HDI) values. The spider chart represents the relative abundances (average values of the two years) of each taxonomic group, in each month (colors) and in each size-fraction (cardinal points in the sphere). *Synechococcus* and *Rhodobacterales* have contributions to community composition in the different size classes that are relatively stable throughout the year, whereas SAR11 and *Planctomycetes* vary significantly their distributions through the year (see section “Materials and Methods” and Table 1 for details).

The relative abundances of each of these taxonomic groups over time (Supplementary Figure S7) revealed that, while some maintained their structure across size-fractions, others did not. We devised a way of quantifying, for each taxonomic group, the degree of variation over time of their distribution across size-fractions, named as Heterogeneous Distribution Index, or HDI (Table 1). Calculated for the various taxonomic groups considered, it varied from a value of ca. 2 for those groups that changed little their enrichment over time (i.e., maintained preferences for a given size-fraction), such as *Flavobacteria*, *Synechococcus*, *Rhodobacterales*, *Oceanospirillales*, or *Verrucomicrobia*; to a value of ca. 6 for those groups that presented very different enrichments in each size-fraction at different times of the year, such as SAR11, *Planctomycetes* or SAR116 (Figure 6). Finally, the “monthly HDI for the whole community,” which represents the average HDI of all taxonomic groups, was in general higher during the warmer months and lower during the colder months, indicating that during periods of higher temperatures, the community as a whole presented larger variability in their distribution across the size classes.

**TABLE 1:** Heterogeneous Distribution Index (HDI) values for each taxonomic group and for each month.

		Months												
Category	Taxonomy	January	February	March	April	May	June	July	August	September	October	November	December	Average
*A*	SAR116 clade *(Alphaproteobacteria)*	12.38	4.35	9.59	7.69	7.23	6.07	4.83	4.17	7.8	6.15	5.47	5.12	6.7
	SAR11 clade *(Alphaproteobacteria)*	5.56	7.17	9.41	1.34	7.15	6.2	3.13	9.34	16.31	5.08	7.55	3.9	6.8
*B*	*Synechococcus (Cyanobacteria)*	2.1	3.43	2.26	1.79	3.73	1.35	2.22	1.8	2.78	1.64	2.32	1.42	2.2
*C*	*Actinobacteria*	5.27	2.35	1.76	7.17	3.07	2.6	3.24	2.61	1.8	3.39	3.23	1.4	3.2
	*Deferribacteres*	6.27	1.63	4.78	3.75	9.13	8.37	8.46	5.21	4.47	3.44	4.79	2.69	5.3
	*Oceanospirillales (Gammaproteobacteria)*	2.01	1.03	2.03	3	8.74	1.14	2.34	3.54	1.14	1.79	1.52	1.3	2.5
*D*	*Rhodobacterales (Alphaproteobacteria)*	6.18	2.01	1.15	3.37	1.81	3.49	3.15	1	2.61	2.25	2.84	1.89	2.6
	*Alteromonadales (Gammaproteobacteria)*	2.57	2.39	3.19	2.62	3.12	3.46	3.45	3.92	1.92	2	5.63	3.23	3.1
	*Cytophagia (Bacteroidetes)*	7.23	1.72	4.03	5.77	3.83	4.75	7.02	8.85	3.03	2.51	5.15	4.04	4.8
	*Vibrionales (Gammaproteobacteria)*	6.75	8.6	2.09	4.21	4.57	3.38	13.47	6.5	3.68	2.03	5.21	4.78	5.4
	*Sphingobacteriia (Bacteroidetes)*	2.18	2.24	4.46	9.59	2.89	3.38	4.96	7.24	2.55	1.37	3.38	2.72	3.9
	*Firmicutes*	1.28	1.71	5.11	5.93	2.14	5.02	4.68	3.92	2.24	1.65	3.5	1.9	3.3
	*Planctomycetes*	3.67	3.37	7.17	1.84	5.66	17.97	17.44	2.76	4.02	4.18	3.93	2.16	6.2
	*Verrucomicrobia*	1.99	1.96	3.85	4.54	1.82	1.37	2.79	1.02	1.73	1.02	4.17	1.04	2.3
	*Rhizobiales (Alphaproteobacteria)*	1.11	3.09	4.05	2.52	2.53	2.94	3.55	1.8	2.01	3.99	2.01	1.37	2.6
	*Deltaproteobacteria*	1.6	4.9	1.77	2.03	5.61	4.21	8.92	2.46	2.86	3.1	6.47	4.49	4
	*Flavobacteria (Bacteroidetes)*	1.05	1.4	1.71	4.37	1.43	1.62	3.06	2.09	0.93	0.7	2.65	1	1.8
	Average all groups together	3.96	3.04	3.89	4.14	4.23	4.48	5.54	3.87	3.55	2.71	4.28	2.54	3.9

*Annual averages of HDI values are also included (see section “Materials and Methods” for details related to the calculation of HDI). Taxonomic groups are classified in 4 categories that correspond to their preference for small or large size-fractions (see section “Results” for details of this classification).*

## Discussion

Bacterial diversity and community assembly in the particulate matter continuum vary spatially, as has been shown before in the Mediterranean (Mestre et al., 2017b) or elsewhere (Mestre et al., 2018). Yet, we know little about how diversity and the communities present in the particulate matter continuum vary temporally. Recently (Yung et al., 2016), reported on general temporal variations using a multiple size-fractionation scheme. Now, we use a larger data-set, and we focused on examining the seasonal changes. We specifically test whether bacteria (at individual taxonomic groups and at the community level) inhabiting the different size-fractions display repeatable changes over the year and what environmental key drivers influence their recurrent seasonal patterns.

### Seasonal Variability of Bacterial Communities in the Particulate Matter Continuum

Previous studies have described that, in temperate sites, the intra-annual variability of community composition of FL bacteria can have two distinct dynamics: either gradual changes throughout the year (Schauer et al., 2003) or non-continuous and rapid transitions between warm and cold months (Ward et al., 2017). Studies analyzing the temporal variability of ATT bacteria are scarce and while supporting the idea of different FL and ATT prokaryotic communities, reached contrasting conclusions with regard to which of both communities exhibit larger changes over time (Selje and Simon, 2003; Mohit et al., 2014; Yung et al., 2016). Here, we observed that the community assembly of both the FL and the various ATT size-fractions were different, varied over time, and this variation was gradual throughout the year for all size-fractions. In our integrative effort in sampling and data analysis, we observed that community assembly presented two main configurations, corresponding to colder and warmer months. We also observed a systematic oscillation between the two main configurations (i.e., winter and summer) and this gradual oscillation has also been detected in the same area using a 10 years’ time series of unicellular pico- and nano- eukaryotes (Giner et al., 2019). In addition, we observed that the fraction that showed less variability in community composition over the year was the smallest, whereas the larger the size-fraction was the more variable community over time. This result suggests that FL communities have a more homogeneous niche along the year than their ATT counterparts. Variability in community composition in larger size-fractions may be linked to the annual variability of particle composition, that may be chemically and ecologically more variable than the dissolved phase. Yet, we know little of the intra-annual variability of particle characteristics in Blanes Bay, nor from elsewhere.

Particles are heterogeneous and can be originated in-situ [e.g., plankton cells and derived particles such as transparent exopolymeric particles (TEPs) and fecal pellets] or arrive from allochthonous sources such as sediment resuspension, atmospheric deposition and terrestrial runoff (e.g., Guerzoni et al., 1999; Ferré et al., 2005; Lucea et al., 2005). In Blanes Bay, the water column is completely mixed during most of the year except summer (Vila-Costa et al., 2007), and this mixing may contribute to sediment resuspension, especially during periods of windstorms and waves (Guadayol et al., 2009). In summer, the water is in general stratified, preventing sediment resuspension, but dust atmospheric deposition has the annual maximum values in this area (Querol et al., 2009). How this input of allochthonous particles affect specifically attached bacterial communities in Blanes Bay is unknown, but there are studies in the same area analyzing their effect over FL bacteria, and these studies show that dust addition experiments contribute little to changes in FL microbial community composition (Lekunberri et al., 2010; Laghdass et al., 2011; Pulido-Villena et al., 2014; Marín-Beltrán et al., 2019). In Blanes, zooplankton, phytoplankton, and unicellular nanoeukaryotes display clear changes of abundance and diversity throughout the year (Calbet et al., 2001; Gasol et al., 2016; Nunes et al., 2018; Giner et al., 2019) and particles produced by phytoplankton such as TEPs (Passow, 2002) also exhibit a seasonal cycle (Ortega-Retuerta et al., 2018). Some bacteria are physically linked to planktonic communities (e.g., Grossart et al., 2005; Tang et al., 2010; DeCorte et al., 2014; Seymour et al., 2017) or their derived particles such as fecal pellets (Hansen et al., 1996) or TEPs (Zäncker et al., 2019). Consequently, it is very likely that annual variations in particle-associated bacterial community structure might in part be related to the seasonal changes in phytoplankton, zooplankton, and other unicellular eukaryotes to which bacteria can attach and in some other cases they might be associated to the input of allochthonous particles such as those that are resuspended from the sediment or that arrive by atmospheric deposition.

The key drivers of bacterial community structure in all size-fractions were sea surface temperature and day length, and the same was observed in previous studies analyzing only the FL bacteria in temperate sites (Pinhassi and Hagström, 2000; Pinhassi et al., 2006; Andersson et al., 2010), and elsewhere (Gilbert et al., 2012; Chow et al., 2013; Ward et al., 2017). Remarkably, together with temperature and day length, other measured variables played a role in determining bacterial community structure in different size-fractions. For example, small size-fractions were highly influenced by dissolved PO_4_ concentration, which is a limiting nutrient for bacterial growth in the NW Mediterranean (Sala et al., 2002; Pinhassi et al., 2006), while chlorophyll and turbidity (i.e., proxies of the quality and quantity of particles in the water column) were important drivers for the attached community, something that has been suggested before (Simon et al., 2002). Conversely, Yung et al. (2016) observed no relationship between any of their environmental variables (bulk-water) and bacterial composition in larger size-fractions, but they did not measure the same variables as we did. Yung et al. (2016) argued that FL bacteria are likely more responsive to bulk-water properties than particle-attached bacteria, and that bacteria on particles may be protected from environmental conditions or could respond to factors that they had not measured. Our data indicates that ATT bacterial communities are not completely isolated from bulk-water properties (as they were driven e.g., by chlorophyll and turbidity) and thus we can conclude that ATT bacteria are influenced by both the properties of the waters surrounding the particles and by the specific niches occurring in different size-fractions (Mestre et al., 2017a).

### A Harmonic Seasonal Oscillation of Bacterial Diversity in the Particulate Matter Continuum

Another detected seasonal pattern is that bacterial diversity in all size-fractions increased during the warmer months (from May to October), showing a maximum between October and November, and decreased during the cold months (from November to April). A higher diversity towards colder months had already been reported in temperate sites for the FL fraction (García et al., 2015; Rieck et al., 2015), aerobic anoxygenic phototrophic bacteria (AAPs) (Ferrera et al., 2013), Archaea (Galand et al., 2010) and unicellular pico- and nano-eukaryotes (Giner et al., 2019). Although the highest values in the diversity of FL bacteria, archaea, AAPs, unicellular eukaryotes, and bacteria in all size-fractions were very close in time, they were not exactly coincident, evidence that different environmental conditions drive the phenology of each planktonic group.

Interestingly, when we fit a harmonic model to the temporal variability of the various diversity measures (alpha, beta, and gamma bacterial diversity), we observed that the model presented an annual pattern similar to that of day length and temperature. We, moreover, observed that all these variables had their annual maximum during the warmer months, and their minimum during the colder months. Different maximum peaks were separated by a small time lag. Annual harmonic oscillations with maxima during the warmer months were also observed at the same area for various components of the dissolved organic matter pool, with dissolved organic carbon (DOC) accumulating and becoming more refractory in late summer (Romera-Castillo et al., 2013). Also in the same sampling site, TEPs peaked during early summer, indicating that TEP-enriched particles accumulate in surface waters during the stratified periods (Ortega-Retuerta et al., 2018). Thus, we could hypothesize that the progressive accumulation of dissolved and particulate complex organic molecules during the warmer months may likely promote the increase in bacterial diversity within each size-fraction by creating new niches within the particulate matter continuum. Furthermore, the sequence of harmonic events reveals that all oscillations may be linked: day length directly affects seawater temperature, which produces summer water column stratification, preventing the flux of new inorganic nutrients into the bay. This stratification would favor the accumulation of DOC and TEPs during summer that would in turn influence the composition of the particles, increasing the diversity of bacteria present in the different size-fractions during the warmer months. Still, we lack studies describing the annual variation in the composition of the particulate matter, as well as studies describing the mechanistic processes modifying the POC and DOC pools. We will need this information to better disentangle the links among the harmonic oscillations occurring along the year.

### Some Taxonomic Groups Can Change Seasonally Their Preference for a Particular Size-Fraction

Most abundant bacterial taxa can colonize several size-ranges of the particulate matter continuum (e.g., from 0.2 to 200 μm) and, depending on their distribution along this spectrum, bacterial taxa can be classified into two major groups: those with preference for small size-fractions, and those with preference for larger size-fractions (see details in Mestre et al., 2017a). Still, a detailed analysis of how taxa inhabiting the particulate matter continuum vary through time was lacking. First, we evaluated if all taxa preference for certain size-fractions was consistent throughout the year or, in contrast, whether they varied their preference to some extent. For this, we devised a way of quantifying this variability: the HDI (Heterogeneous Distribution Index). The HDI tells us, for each taxonomic group, the degree of consistency over time of their enrichment across size-fractions. Most of the taxonomic groups presented low values of HDI, indicating that the preference for a given size-fraction is constant over time for most taxa, but three of them (Planctomycetes, SAR11 and SAR116) presented elevated values, revealing that these taxa were more abundant in a size fraction at some parts of the year and in another size fraction during other parts of the year.

Our results reveal that the size-fractionation scheme together with the HDI are useful to describe the temporal variability of a given taxonomic group. For example, in the case of *Synecchococcus* sp. we found that their relative abundances were quite high all over the year with increases during the warmer months (as previously reported in Alonso-Sáez et al., 2007; Gasol et al., 2016; Nunes et al., 2018) and that *Synecchococcus* sp. had higher abundances in smaller size-fractions (0.8–3.0 μm) but was depleted or absent in the smallest size-fraction (0.2–0.8 μm) (as previously reported in Mestre et al., 2017a). We can add that *Synechococcus* sp. had low HDI values, i.e., this preference for small sizes (0.8–3.0 μm) is consistent through the whole seasonal cycle. Other groups that maintained their structure along the size-fractions over time (i.e., with low HDI values) were *Flavobacteria* and *Rhodobacterales.* Both taxonomic groups were abundant along the year (5–20% of total community abundance), especially in the warmer months, and appeared especially in larger size-fractions. We observed that *Flavobacteria* and *Rhodobacterales* presented strong variations in abundances from one month to the other, probably responding to monthly changes in the composition of the particles, as it is known that both groups can respond rapidly to algal blooms (Buchan et al., 2014; Needham and Fuhrman, 2016). Still, despite their rapid monthly changes in abundances, both taxa always maintained their preference for larger size-fractions.

Conversely, SAR11 is an example of a taxonomic group that eventually varies its enrichment in small size-fractions over time (i.e., high HDI): as a general trend, we observed that SAR11 was more dominant in the smallest size-fraction. This general rule changed during the cold period, when the presence of SAR11 in particles increased. Moreover, the diversity of SAR11 (number of OTUs) increased during the cold period (Supplementary Figures S8, S9), in agreement with previous observations (Salter et al., 2014). This reveals that during winter there is a high diversification of SAR11, when some of the clades/ecotypes are strongly adapted to the attached lifestyle. In agreement with our data, SAR11 has been described mostly as FL bacteria (Giovannoni and Vergin, 2012), but with some ecotypes adapted to particles (Allen et al., 2012). Here we observed that SAR11 attached to particles concurred with the typical late-winter phytoplankton bloom of the NW Mediterranean Sea (Duarte et al., 1999; Gasol et al., 2016), which suggests that the SAR11-attached populations might respond to pulses of particulate matter derived from phytoplankton. Hunt et al. (2008) demonstrated that closely related strains coexisting in the same site can inhabit different size-fractions, thus, showing resource partitioning and sympatric differentiation among closely related bacteria. We hypothesize that the same ecological differentiation may occur within the SAR11 clade, a group that presents high microdiversity (García-Martínez and Rodríguez-Valera, 2000; Brown and Fuhrman, 2005).

## Conclusions

Our multiple size-fractionation approach applied to a temporal time series indicates that the bacterial communities present in each size-fraction changed monthly, and that communities in larger size-fractions are the more variable over time. These temporal changes were gradual throghout the year and were closely related to the variation in day-length and sea surface temperature. Further, specific environmental factors influenced certain size-fractions, as e.g., smaller size-fractions were more influenced by PO_4_, whereas attached communities were more driven by turbidity. Diversity (including all fractions) was higher at the end of summer along with a higher differentiation of the communities present on different particles. As a general rule, most abundant taxonomic groups retained their preference for certain size-fractions along the year. Yet, certain taxa may change their preferences over time. For example, SAR11 ecotypes preferred smaller size-fractions in summer and larger size-fractions in winter. Summarizing, the study of bacteria in several size-fractions in a time series generates a broad vision of the seasonal dynamics of bacterial communities, and reinforces the importance of taking the particulate matter continuum into account to describe and unveil in a more comprehensive way the ecology of aquatic microorganisms in their complex planktonic microbial habitats.

## Data Availability Statement

The raw sequence data have been deposited at the National Center for Biotechnology Information (NCBI). The data can be accessed online (https://www.ncbi.nlm) with the code PRJNA345534.

## Author Contributions

MM, MS, and JG contributed to the conception and design of the study. MM extracted the DNA, processed genomic sequences and analyzed data. JH performed harmonic analysis and prepared the associated figure and text. JG developed the Heterogeneous Distribution Index and prepared the associated table, figure, and text. MM interpreted the results and wrote this article. All authors wrote sections of the manuscript and contributed to manuscript revisions.

## Conflict of Interest

The authors declare that the research was conducted in the absence of any commercial or financial relationships that could be construed as a potential conflict of interest.

## Footnotes

1 www.researchandtesting.com

2 www.r-project.org
