# Supplementary material for: Seasonal Variation of Bacterial Diversity Along the Marine Particulate Matter Continuum
Source: Front Microbiol. 2020 Jul 21;11:1590. doi: 10.3389/fmicb.2020.01590 (PMC7385255; doi:10.3389/fmicb.2020.01590)
Supplement: Supplementary file 1 [file Image_1.pdf]

Supplementary Material of the paper:

# Seasonal variation of bacterial diversity along the marine particulate matter continuum

By Mireia Mestre, Juan Höfer, M. Montserrat Sala, Josep M. Gasol (2020)

Contact: Mireia Mestre
Email: mireia.mestre.martin@gmail.com

This PDF file includes:

Supplementary Figure S1
Supplementary Figure S2
Supplementary Figure S3
Supplementary Figure S4
Supplementary Figure S5
Supplementary Figure S6
Supplementary Figure S7
Supplementary Figure S8
Supplementary Figure S9

Supplementary Table S1
Supplementary Table S2
Supplementary Table S3

Supplementary Methods S1
Supplementary Methods S2

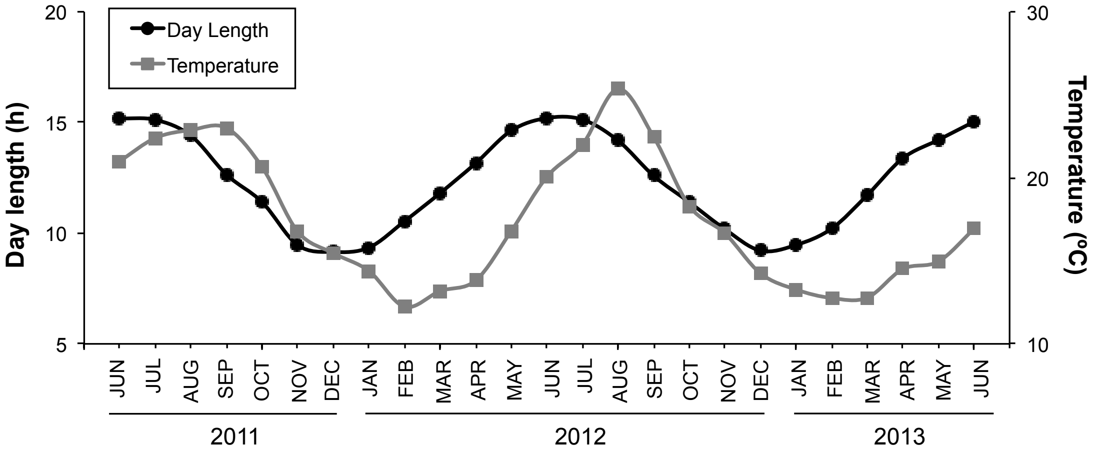


**Supplementary Figure S1.** Day length and sea surface temperature variability in Blanes Bay Microbial Observatory from June 2011 to June 2013.

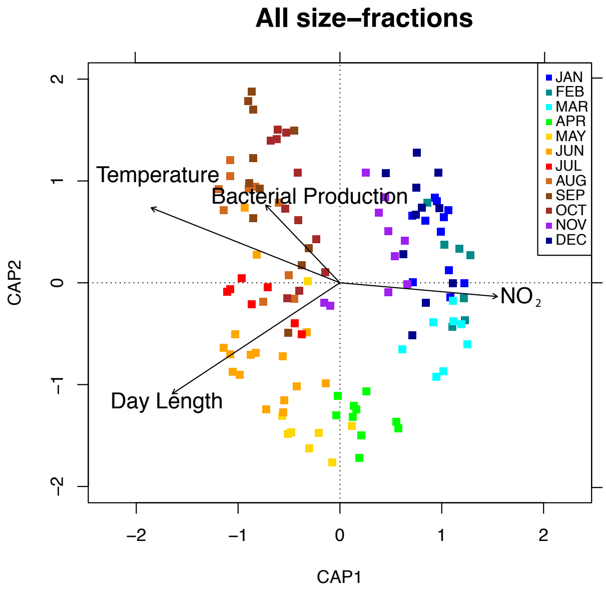


**Supplementary Figure S2.** Distance-based redundancy analysis (dbRDA) representing all samples (dots) and the main environmental parameters (arrows). See Material and Methods for details.

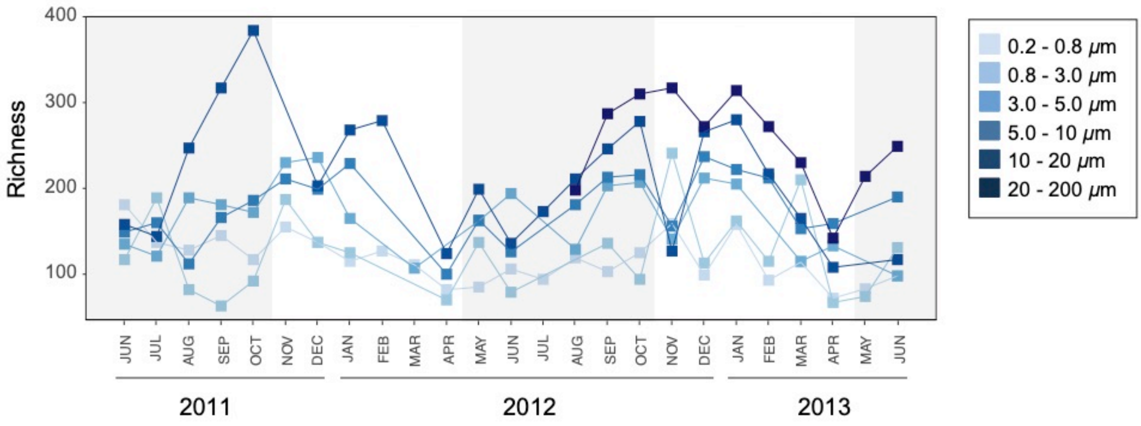


**Supplementary Figure S3.** Richness (number of OTUs) in each size-fraction and in each month from June 2011 to June 2013. Gray background corresponds to the warm period and white background corresponds to the cold period (see Results for more information about this division).

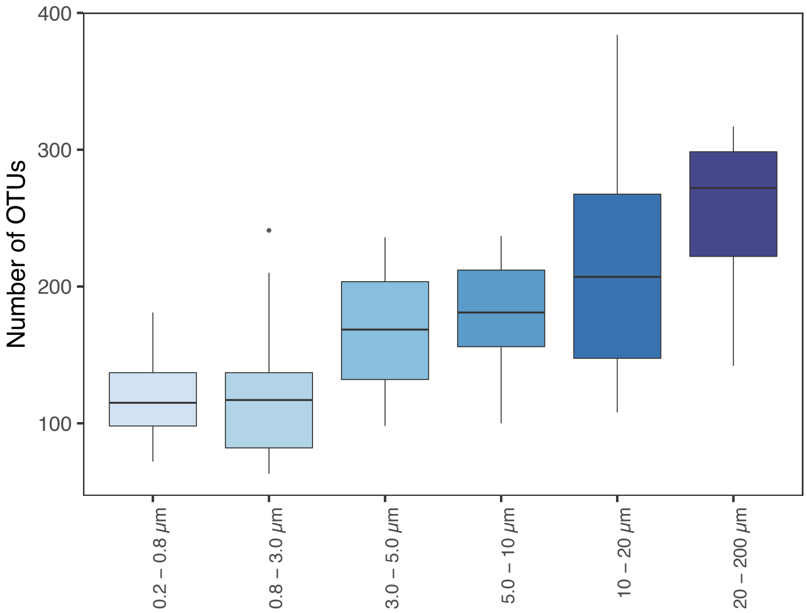


**Supplementary Figure S4.** Box-plot of the richness (number of OTUs) per size-fraction. Upper and lower lines correspond to the first and third quartile of the distribution of values. The median values are shown with horizontal black darker lines. Outliers are displayed as dots.

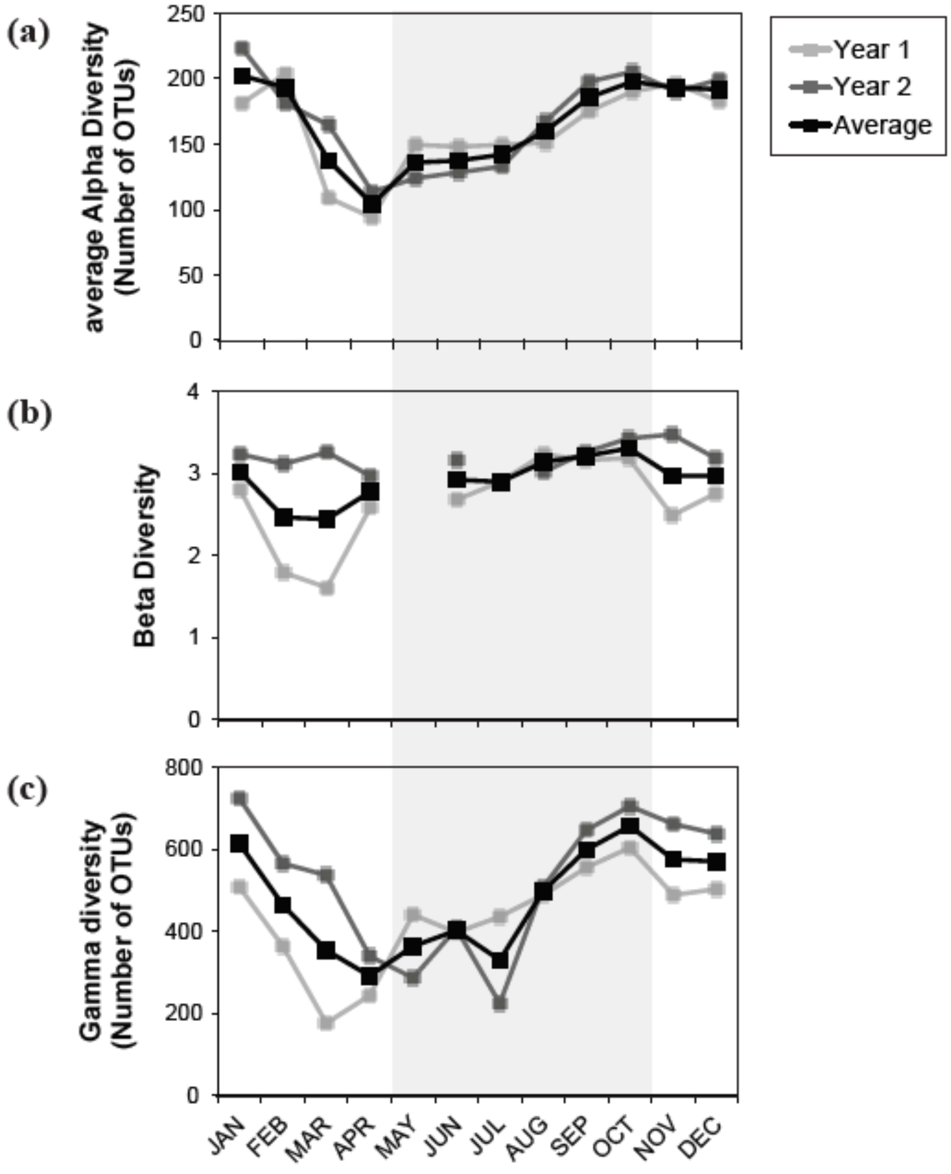


**Suplementary Figure S5:** Average alpha-**(a)**, beta-**(b)** and gamma-**(c)** diversity for each month. Values of each year and its average are represented separately. Gray background corresponds to the warm period and white background corresponds to the cold period (see Results for more information about this division).

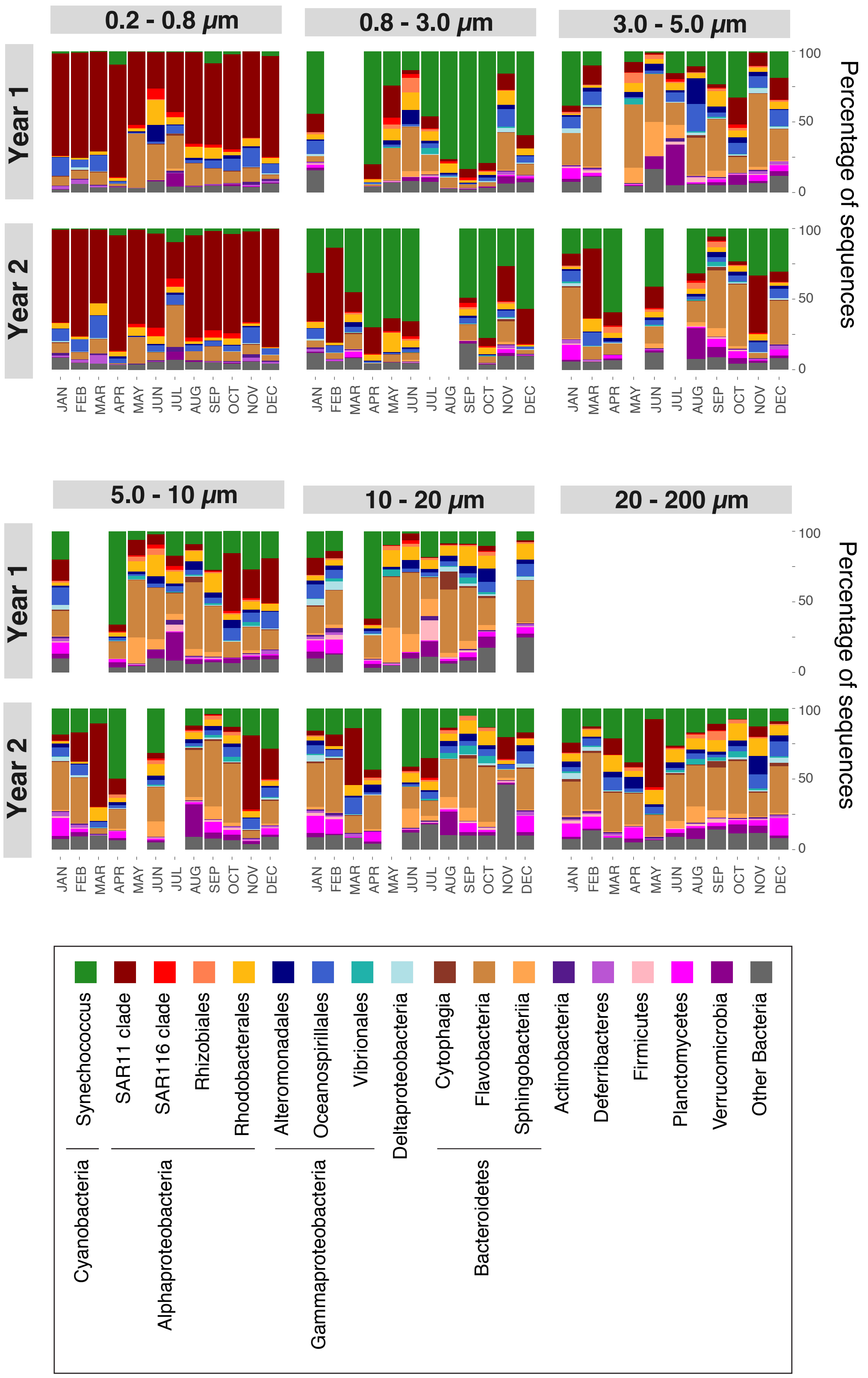


**Supplementary Figure S6.** Taxonomic composition in each month and in each size-fraction. Only taxonomic groups with >1% in abundance are represented, and the remaining taxonomic groups are pooled together as "other bacteria". Abundances are expressed as percentages of the total number of sequences in each size-fraction (for more details see Material and Methods).

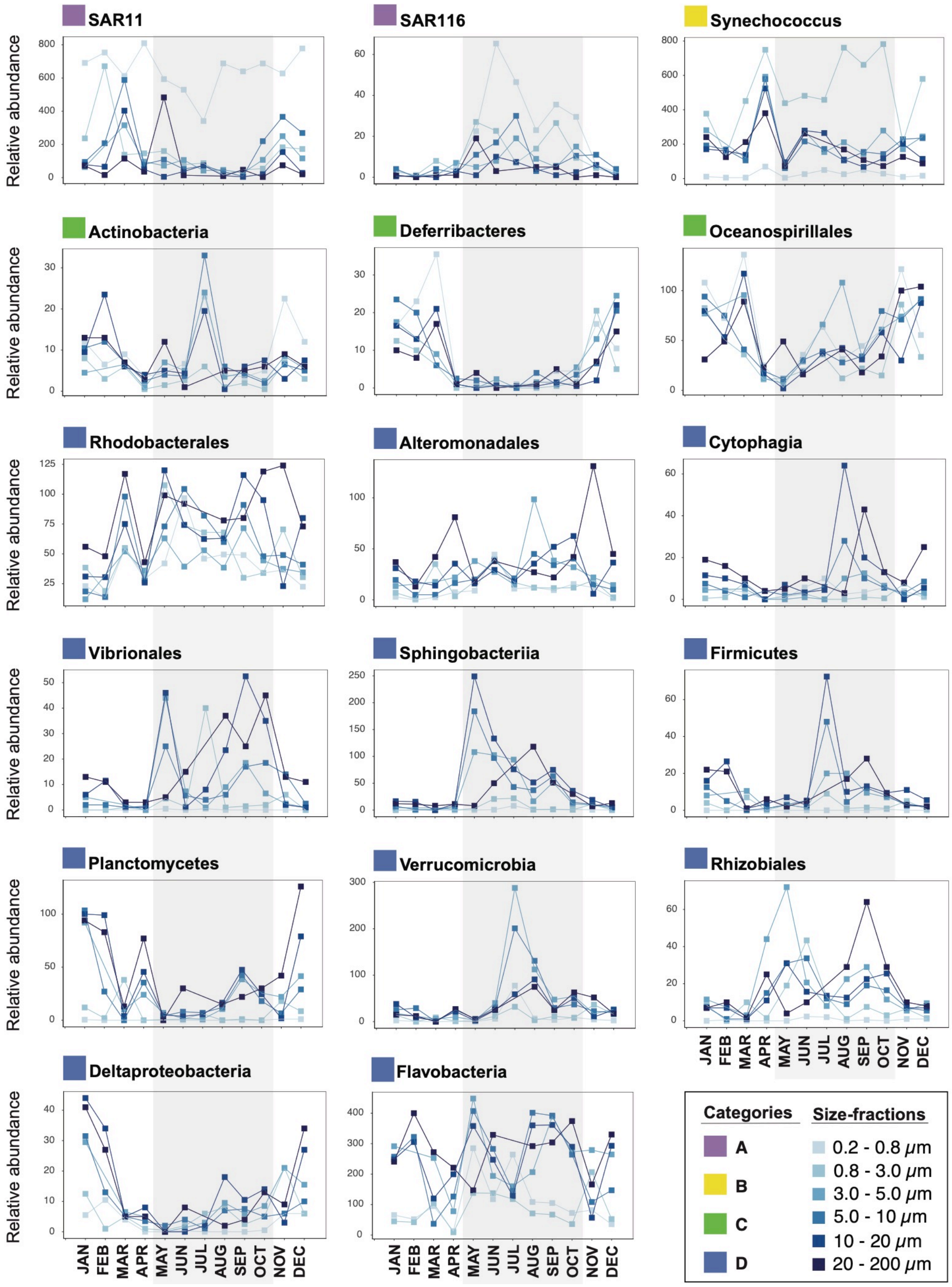


**Supplementary Figure S7:** Relative abundances (average values of both years) of the most abundant taxonomic groups in each size-fraction and from January to December. Taxonomic groups were grouped into 4 categories, following the classification of Mestre *et al*. (2017a): (a) taxonomic groups enriched in the small size-fractions; (b) taxonomic groups enriched in larger size-fractions, but depleted or absent in the smallest one (0.2–0.8 μm); (c) taxonomic groups that do not present enrichment in relation to the size-fraction; and (d) taxonomic groups enriched in larger size-fractions. Gray background corresponds to the warm period and white background corresponds to the cold period of the year (see Results for more information about the periods).

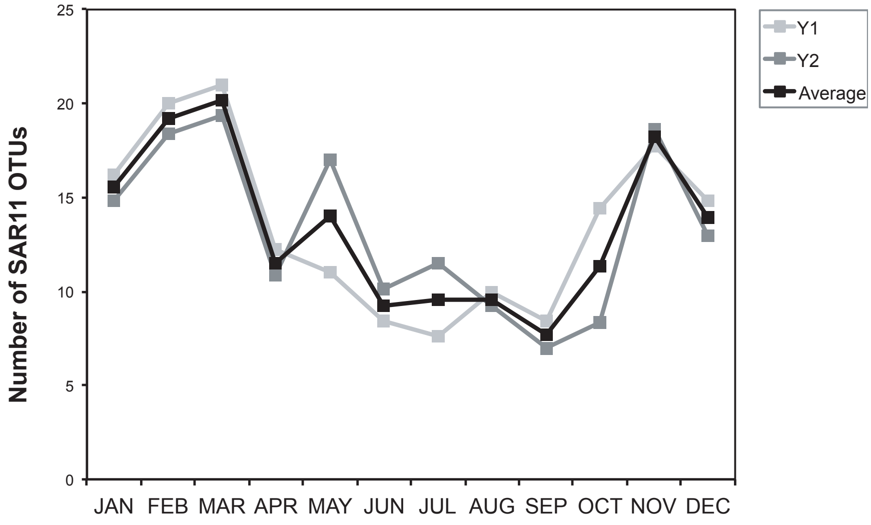


**Supplementary Figure S8:** Number of SAR11 OTUs in each month, calculated as an average number of OTUs among all size-fractions. Values of each year and its biannual average are represented separately.

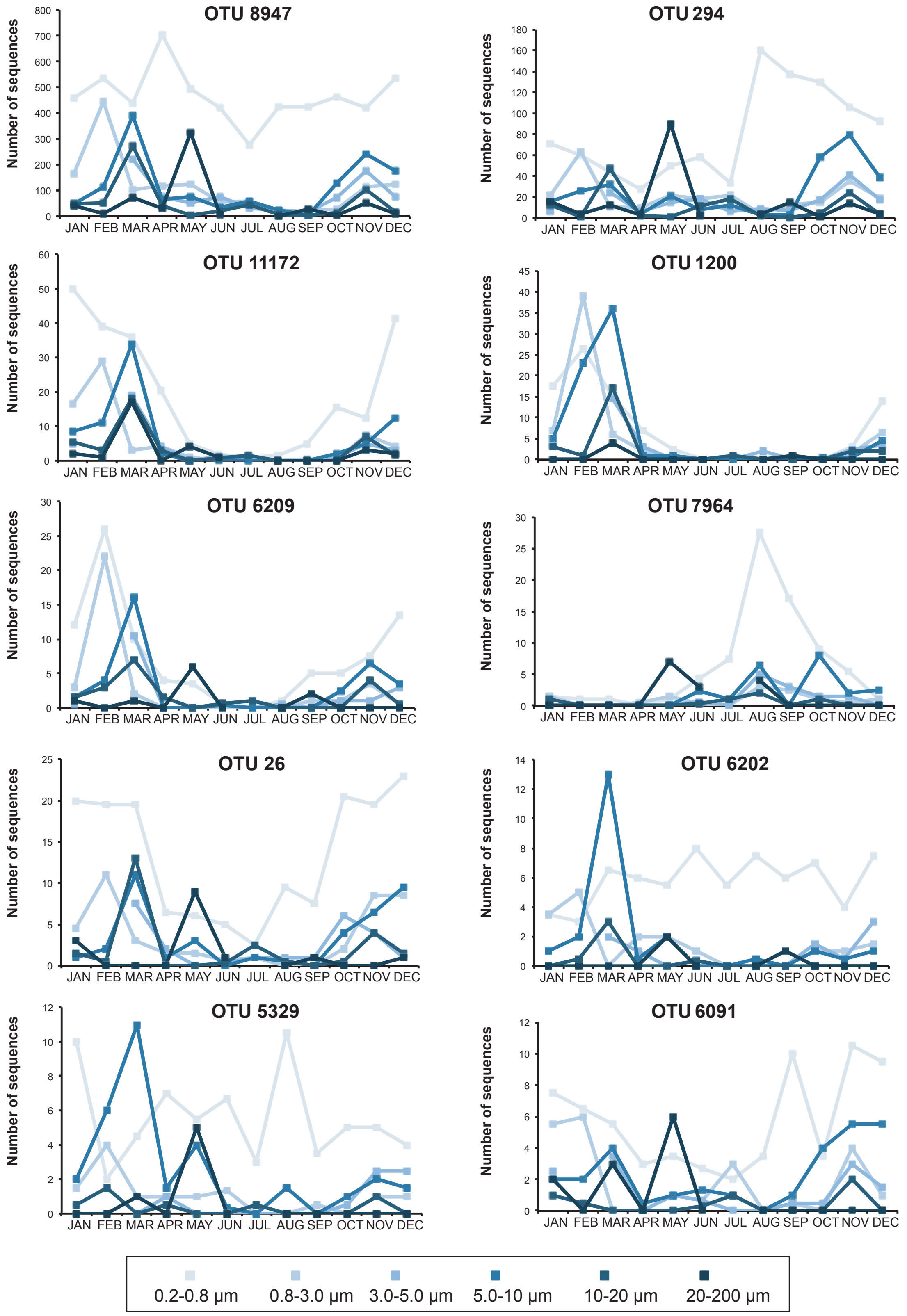


**Supplementary Figure S9:** Average number of sequences (average values of both years) of the most abundant SAR11 OTUs (accounting for approximatelly the 95% of all the SAR11 sequences) in each size-fraction and from January to December. SAR11 OTUs were obtained from the rarefied OTU table.

**Supplementary Table S1.** Correlation coefficients between each environmental variable and the dbRDA coordinate axes.

| | **dbRDA1** | **dbRDA2** | **dbRDA3** | **dbRDA4** |
|---:|---:|---:|---:|---:|
| **Day Length** | -0.82 | -0.54 | -0.03 | -0.18 |
| **Temperature** | -0.92 | 0.37 | 0.12 | -0.05 |
| **$NO_2$** | 0.77 | -0.07 | -0.07 | -0.63 |
| **Bacterial Activity** | -0.36 | 0.38 | -0.83 | 0.18 |

**Supplementary Table S2.** Correlation coefficients between each environmental variable and the dbRDA coordinate axes, for each size-fraction separately.

| Size-fraction | Variable | dbRDA1 | dbRDA2 | dbRDA3 | dbRDA4 | dbRDA5 | dbRDA6 | dbRDA7 | dbRDA8 |
|---|---|---|---|---|---|---|---|---|---|
| **0.2 - 0.8 µm** | **Day length** | -0.81 | -0.52 | -0.19 | 0.21 | | | | |
| | **Temperature** | -0.93 | 0.36 | 0.03 | 0.04 | | | | |
| | **$PO_4$** | 0.32 | 0.29 | -0.9 | 0.09 | | | | |
| | **$SiO_2$** | 0.61 | 0.02 | -0.37 | 0.7 | | | | |
| **0.8 - 3.0 µm** | **Day length** | -0.84 | -0.47 | -0.24 | 0.09 | -0.07 | | | |
| | **Temperature** | -0.84 | 0.48 | 0.02 | 0.23 | -0.11 | | | |
| | **Chlorophyll *a*** | 0.41 | 0.18 | -0.47 | 0.02 | 0.76 | | | |
| | **$PO_4$** | 0.34 | 0.17 | -0.75 | 0.03 | -0.55 | | | |
| | **$NO_2$** | 0.77 | -0.39 | 0.08 | 0.45 | -0.19 | | | |
| **3.0 - 5.0 µm** | **Day length** | -0.77 | -0.63 | -0.09 | 0.08 | | | | |
| | **Temperature** | -0.95 | 0.22 | -0.08 | -0.2 | | | | |
| | **$SiO_2$** | 0.49 | -0.13 | -0.85 | 0.13 | | | | |
| | **Bacterial activity** | -0.39 | 0.5 | 0.05 | 0.78 | | | | |
| **5.0 - 10 µm** | **Day length** | -0.8 | 0.46 | -0.2 | 0.34 | | | | |
| | **Temperature** | -0.88 | -0.17 | 0.43 | -0.09 | | | | |
| | **Secchi depth** | -0.74 | -0.51 | -0.38 | 0.22 | | | | |
| | **Bacterial abundance** | -0.03 | 0.61 | -0.17 | -0.77 | | | | |
| **10 - 20 µm** | **Day Length** | -0.8 | 0.56 | 0.18 | 0.09 | | | | |
| | **Temperature** | -0.97 | -0.25 | -0.06 | 0.01 | | | | |
| | **Secchi depth** | -0.5 | -0.29 | 0.81 | -0.06 | | | | |
| | **$SiO_2$** | 0.59 | 0.04 | -0.23 | 0.77 | | | | |
| **20 - 200 µm** | **Day length** | -0.79 | 0.54 | 0.09 | -0.16 | 0.09 | -0.01 | 0.05 | -0.22 |
| | **Temperature** | -0.8 | -0.33 | -0.22 | 0.07 | 0.42 | -0.02 | -0.11 | -0.04 |
| | **Secchi depth** | -0.72 | -0.15 | 0.51 | 0.03 | 0.07 | -0.37 | -0.23 | -0.04 |
| | **Salinity** | 0.42 | -0.41 | -0.22 | -0.03 | 0.72 | 0.11 | -0.09 | 0.25 |
| | **Chlorophyll *a*** | 0.76 | 0.48 | 0.34 | 0.18 | 0.08 | 0.08 | 0.04 | 0.19 |
| | **$NO_2$** | 0.76 | 0 | 0.26 | -0.27 | 0.02 | -0.43 | 0.31 | -0.03 |
| | **$SiO_2$** | 0.79 | 0.22 | -0.09 | 0.29 | -0.06 | 0.4 | 0.02 | -0.27 |
| | **Bacterial abundance** | 0.1 | 0.42 | -0.66 | -0.07 | -0.53 | -0.08 | -0.29 | 0.04 |

**Supplementary Table S3.** Fitted parameters (±standard error) of the harmonic analyses of the studied variables: annual mean (*b1*), amplitude (*b2*) and diphase (*b3*). Coefficients *b1* and *b2* are expressed in the units of each variable and *b3* is expressed in day of the year (Ordinal date). $R^2$, determination coefficient; n, number of data of each time series; p-value, significance of the linear regression between the measured and the modeled data.

| | b1 (annual mean) | b2 (amplitude) | b3 (seasonal maximum) | $R^2$ | p-value | n |
|---|---|---|---|---|---|---|
| Day length | 12.22 ± 0.015 | -2.96 ± 0.021 | 169.81 ± 0.008 | 0.998 | <0.001 | 25 |
| Temperature | 17.51 ± 0.232 | 5.42 ± 0.334 | 222.25 ± 0.059 | 0.920 | <0.001 | 25 |
| Alpha | 166.40 ± 4.034 | 38.66 ± 5.633 | 308.682 ± 0.149 | 0.669 | <0.001 | 25 |
| Beta | 2.89 ± 0.104 | -0.32 ± 0.153 | 249.82 ± 0.444 | 0.130 | 0.043 | 25 |
| Gamma | 486.94 ± 21.513 | -150.60 ± 30.611 | 293.23 ± 0.201 | 0.505 | <0.001 | 25 |

**Supplementary Methods S1: Measurement of environmental parameters.**

To determine prokaryotic abundance on the particles, seawater was fixed with glutaraldehyde (final concentration 1%) and distinct volumes were filtered through black polycarbonate membrane filters (Poretics) of 5 different pore-sizes: 0.2 µm (5 mL), 0.8 µm (20 mL), 3.0 µm (150 mL), 5.0 µm (150 mL), 10.0 µm (150 mL). Before finishing filtering all the volume, the last 5 mL of each sample were maintained for 5 min with 50 µL of DAPI dye (0.5 mg ml-1) in the dark. The filters were placed on microscope slides and with immersion oil (Type-F, Olympus). DAPI positive cells were enumerated by epifluorescence microscopy (Olympus BX61 epifluorescence microscope).

Temperature and salinity were measured with a CTD probe (SD2014, SAIV A/S). Chlorophyll a was measured according to the procedure of Yentsch and Menzel (1963). Bacterial heterotrophic activity was estimated using the $^{3}$H-leucine incorporation method (Kirchman et al., 1985). Inorganic nutrients were analyzed using a CFA Bran Luebbe autoanalyser following the methods described by Hansen and Koroleff (1999). Samples for total organic carbon (TOC) determinations were collected in 10 mL precombusted (450 ºC, 24 h) glass ampoules. After acidification with 50 µL 25% $H_3PO_4$ to pH<2, the ampoules were heat-sealed and stored in the dark at 4ºC until analysis. Analyses were carried out using a Shimadzu TOC-CSV organic carbon analyzer. Particulate organic carbon (POC) was measured by filtering 60 mL (four replicates) on pre-combusted GF/F glass fiber filters. The filters were then frozen in liquid nitrogen and kept at –80ºC until analysis. Prior to analysis, the filters were dried at 60ºC for 24 h and exposed to hydrochloric acid vapors for 48 h to destroy inorganic material. They were then analyzed in a Perkin-Elmer 240 C:H:N autoanalyser.

**Bibliography:**

-Hansen HP, Koroleff F. (1999). Determination of nutrients. In: Grasshoff K, Kremling K, Ehrhardt M (eds). Methods of Seawater Analysis. Wiley-VCH: Weinheim, Germany, pp 159–228.

-Kirchman D, Knees E, Hodson R. (1985). Leucine incorporation and its potential as a measure of protein-synthesis by bacteria in natural aquatic systems. Appl Environ Microbiol 49:599–607.

-Yentsch CS, Menzel DW. (1963). A method for the determination of phytoplankton chlorophyll and phaeophytin by fluorescence. Deep-Sea Research 10:221–231.

**Supplementary Methods S2: Computation of the Heterogeneous Distribution Index (HDI).**

We devised a "Heterogeneous Distribution Index" (HDI), to quantitatively evaluate whether a given taxonomic groups maintained or shifted its preferences for a given size-fraction throughout the year. In this algorithm, and for each taxonomic group, the relative abundances of all size-fractions and months were averaged (named as "annual average" of a particular taxonomic groups). Taking the annual averages of a given taxonomic group as a reference, we calculated the deviations that occurred every month and in each size-fraction from that annual average (the absolute difference between a given value and the annual average). Then, the sum of the absolute deviations of all size-fractions in each month for a given taxonomic group was calculated. This relative value was named HDI. It has no units and is a relative measure, being low when all distributions are similar, high when they are very distinct, and equal to 1 if all distributions are identical.

A step by step guide to its calculation follows:

-Step1: For a given sampling (in our case, month) and a given bacterial group, we list the % community composition across size classes (i.e. filters), and calculate the added value (summation) and the relative contribution of each size classes to this total value. This gives the relative importance of each of the filter sizes for each month. E.g. since *Synechoccocus* are 38% of the community in the 0.8-3 μm filter, they have a higher value than in the size fraction 0.2-0.8 μm where they were only 1% of the community.

-Step2: These values are averaged over the different months.

-Step3: The monthly values are standardized to the annual average (i.e. the monthly values of Step1 divided by Step2 average value). A value of e.g. 0.78 here indicates that the value in that particular month and size class is 78% of the yearly average.

-Step4: These values are then averaged over all the months and the deviation of each month to the yearly average computed as Monthly value – yearly average. These are the values in Figure 6.

-Step5: The absolute value of these deviations can be then summed up across filters (size fractions) for a given group, or across months for a given group and size fraction. A low value indicates a relatively constant contribution of the group to community structure across size fractions, or across months. These values are the HDI values which can be further averaged per group or per month (as we do in Table 1) to estimate the relative variability around the mean of the contributions to community structure by group or overall by month.
